# Accuracy of prediction of infarct-related arrhythmic circuits from image-based models reconstructed from low and high resolution MRI

**DOI:** 10.3389/fphys.2015.00282

**Published:** 2015-10-13

**Authors:** Dongdong Deng, Hermenegild Arevalo, Farhad Pashakhanloo, Adityo Prakosa, Hiroshi Ashikaga, Elliot McVeigh, Henry Halperin, Natalia Trayanova

**Affiliations:** ^1^Department of Biomedical Engineering, Institute for Computational Medicine, Johns Hopkins UniversityBaltimore, MD, USA; ^2^Division of Cardiology, Department of Medicine, Johns Hopkins Medical InstituteBaltimore, MD, USA; ^3^Department of Biomedical Engineering, Johns Hopkins UniversityBaltimore, MD, USA

**Keywords:** myocardial infarction, arrhythmia, computational modeling, reentry, MRI reconstruction

## Abstract

Identification of optimal ablation sites in hearts with infarct-related ventricular tachycardia (VT) remains difficult to achieve with the current catheter-based mapping techniques. Limitations arise from the ambiguities in determining the reentrant pathways location(s). The goal of this study was to develop experimentally validated, individualized computer models of infarcted swine hearts, reconstructed from high-resolution *ex-vivo* MRI and to examine the accuracy of the reentrant circuit location prediction when models of the same hearts are instead reconstructed from low clinical-resolution MRI scans. To achieve this goal, we utilized retrospective data obtained from four pigs ~10 weeks post infarction that underwent VT induction via programmed stimulation and epicardial activation mapping via a multielectrode epicardial sock. After the experiment, high-resolution *ex-vivo* MRI with late gadolinium enhancement was acquired. The Hi-res images were downsampled into two lower resolutions (Med-res and Low-res) in order to replicate image quality obtainable in the clinic. The images were segmented and models were reconstructed from the three image stacks for each pig heart. VT induction similar to what was performed in the experiment was simulated. Results of the reconstructions showed that the geometry of the ventricles including the infarct could be accurately obtained from Med-res and Low-res images. Simulation results demonstrated that induced VTs in the Med-res and Low-res models were located close to those in Hi-res models. Importantly, all models, regardless of image resolution, accurately predicted the VT morphology and circuit location induced in the experiment. These results demonstrate that MRI-based computer models of hearts with ischemic cardiomyopathy could provide a unique opportunity to predict and analyze VT resulting for from specific infarct architecture, and thus may assist in clinical decisions to identify and ablate the reentrant circuit(s).

## Introduction

Myocardial infarction (MI), a condition characterized by reduced viability of cardiac myocardium due to insufficient blood supply, is a leading cause of lethal ventricular tachyarrhythmia (VT) worldwide (Go et al., 2014). A promising therapeutic approach is to identify and eradicate the arrhythmogenic substrate through an invasive procedure known as catheter ablation (Delacretaz and Stevenson, 2001). This technique utilizes catheters in an electrophysiological (EP) study that records surface electrical activity to identify the location(s) of the VT reentrant circuits, which are then targeted for ablation. However, an EP study is limited to interrogation of the cardiac surfaces alone, which fails to take into account the complex 3D architecture of the infarct and could lead to missing intramural arrhythmogenic substrates. Additionally, the point by point nature of the technique results in long procedure times that increase risk for complications in patients (Stevenson et al., 2008). These limitations have resulted in a success rate of only 58% for first time VT ablation procedures (Callans et al., 1998). Clearly, there remains a need for novel methodologies to safely and effectively identify the specific MI regions that harbor VT reentrant pathways which can then guide successful post-MI VT ablation.

Recent advances in non-invasive imaging techniques have shown promise in supplementing the information gained from EP studies by providing a priori information on infarct structure (Ashikaga et al., 2007; Dickfeld et al., 2008; Tian et al., 2010; Rutherford et al., 2012). In particular, contrast-enhanced (CE) MRI has become the gold standard for assessing the location, transmurality, and composition of MI (Ashikaga et al., 2007; Perez-David et al., 2011; Fernández-Armenta et al., 2013). Recently, the presence of peri-infarct or border zone surrounding the necrotic scar, also known as gray zone (GZ) based on their appearance as regions of intermediate intensity in the CE-MRI scans, have been shown to correlate with increased risk of post-MI mortality (Yan et al., 2006), increased likelihood of spontaneous clinical VT (Roes et al., 2009), and increased VT inducibility via programmed stimulation (Schmidt et al., 2007). Although CE-MRI is a powerful tool to visualize the complexity of the MI structure, it does not provide any insight into the electrical activity in the heart, particularly the location of the VT reentrant circuits. Additionally, while targeting GZ for ablation could result in increased procedure success, the complex distribution of multiple GZs throughout the heart makes identification of the specific arrhythmogenic substrate difficult (Nayyar et al., 2014).

The limitations of EP studies and non-invasive imaging can be addressed by recent advances in individualized, computational modeling of the heart. Recently, our group and others have demonstrated that models of post-MI hearts reconstructed from high resolution *ex-vivo* MRI can be used to gain insight into the mechanisms of post-MI VT initiation and maintenance (Pop et al., 2011; Rantner et al., 2012; Arevalo et al., 2013). Importantly, these studies confirmed that specific regions of GZ within the complex infarct structure promote reentry initiation and maintenance. Work has also already begun on bringing the technology closer to clinical applicability by reconstructing models from low resolution clinical MRI (Ng et al., 2012; Ashikaga et al., 2013; Ringenberg et al., 2014). However, difficulty in obtaining electrical recordings during VT has made it difficult to fully assess the accuracy of computational models in predicting the morphology and location(s) of VT reentrant circuits. Additionally, it is not known whether models generated from low resolution MRI, with its loss of infarct geometry detail, can still accurately predict post-MI VT.

Thus, the goal of this study is (1) to develop experimentally validated, individualized computer models of infarcted pig hearts based on high resolution images that can accurately predict post-infarction VT morphology and reentrant circuit location and (2) to examine the accuracy of the reentrant circuit location prediction when models of the same hearts are instead reconstructed from low clinical-resolution MRI scans. Determining this accuracy will provide the needed information for the development of patient-specific models from clinical MRI scans and their usilization in predictive simulations regarding infarct-related arrhythmia treatment.

## Methods

### Electrophysiological data and image acquisition

Part of the research in this study utilized experimental data and CE-MRI previously published in the study by Ashikaga et al. (2007). In that study, six domestic pigs underwent MI induction via occlusion of the left anterior descending artery. Approximately 10 weeks post-MI, bipolar electrograms were recorded from 300 to 380 ventricular epicardial sites via a multielectrode sock. VT was then induced via programmed stimulation delivered to a bipolar electrode randomly placed on the epicardium. After the procedure, the hearts were explanted and high resolution *ex-vivo* CE-MRI was acquired. The final image matrix was 256 × 256 × 256, and the image resolution was 0.39 × 0.39 × 0.39 mm^3^. In two of the six pig hearts, the induced VT had chaotic and heterogeneous patterns. These two pig data were excluded from the present study.

In order to mimic typical clinical MRI resolutions, original images were downsampled by eliminating the high-frequency components in the Fourier space of the original images using a 3D rectangular mask. The size of the rectangular mask was calculated as a function of the desired resolution in each dimension (Nishimura, 2010). Following this, an inverse FFT was applied to reconstruct the downsampled images. As a result, the corresponding original and the downsampled images had the same voxel (and matrix) size for the segmentation process. Two levels of downsampling were performed resulting in medium resolution (1.5 × 1.5 × 4 mm^3^) and low resolution (1.5 × 1.5 × 8 mm^3^) image stacks.

### Image segmentation

#### Hi-res images

**Figure 1:**
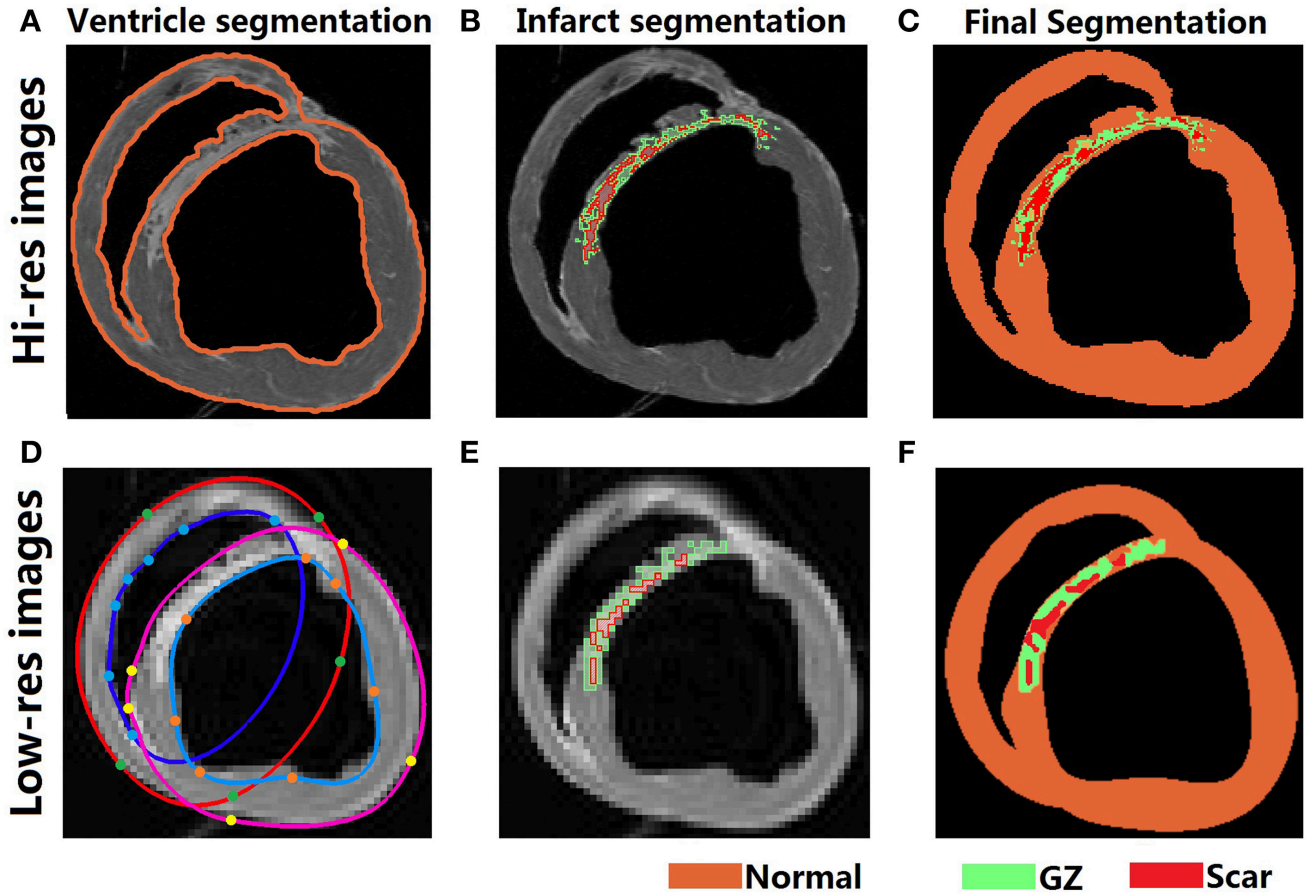
**Segmentation of Hi-res and Low-res images**. **(Top row)** Automatic segmentation of Hi-res ventricular epicardium and endocardium **(A)**, gray level thresholding of infarct into GZ and scar **(B)**, and final segmentation **(C)**. **(Bottom row)** Ventricular segmentation in the low-res images was performed via fitting splines through manually determined landmark points on the boundaries of the epi- and endocardial surfaces **(D)**. Gray level thresholding to segment the GZ and scar was performed similar to Hi-res images **(E)**, and the final interpolated segmattion was shown in **(F)**.

The Otsu threshold method, available in the software package Seg3D (SCI Institute, Salt Lake City, UT, USA), was used to automatically segment the myocardium from the surrounding bath (Figure 1, top row). The myocardium was then further segmented into three tissue types, normal tissue, gray zone (GZ), and scar, using a modified gray-level thresholding algorithm (Ng et al., 2012; Arevalo et al., 2013). First, the region of infarct was manually selected within each slice. Next, the mean and standard deviation (SD) of the voxel intensities in the region outside the infarct were calculated. The gray-level value corresponding to mean + 4 SD was assigned as the upper limit for normal tissue (normal_max_). All voxels with intensities less than normal_max_ were classified as normal tissue. Finally, the range of intensities within the infarct tissue (infarct_range_) was calculated by obtaining the difference between the maximum intensity within the infarct region (infarct_max_) and normal_max_. Voxels with intensity between normal_max_ and (normal_max_ + 50% of infarct_range_) were classified as GZ. Voxels with intensity more than normal_max_ + 50% of infarct_range_ were classified as scar.

#### Med-res and low-res images

The downsampled image stacks were segmented using a previously described segmentation and interpolation method (Prakosa et al., 2014; Ukwatta et al., 2015). Briefly, CardioViz3D (INRIA, Sophia-Antipolis, France), was used to seed points and obtain the surfaces for the epi- and endocardial boundaries of the ventricles (Figure 1, bottom row). The same gray-level thresholding method described in the hi-res image processing section was used to further segment the myocardium. Due to the overall lower range of pixel intensities resulting from the downsampling, the gray-level value corresponding to normal_max_ was calculated from mean + 3 SD as opposed to mean + 4 SD. Calculation of the gray-level thresholds for GZ and scar was done as previously described.

### Mesh generation and fiber assignment

The finite element computational meshes were constructed directly from the segmented images using a previously described approach (Prassl et al., 2009). The procedure preserved the fine geometric details of the ventricles and the different infarct zones. Finally, fiber orientations were assigned in the mesh using a rule-based method (Bayer et al., 2012).

### Modeling of electrophysiological properties

Mathematical description of cardiac tissue was based on the monodomain representation (Plank et al., 2008). The scar was modeled as passive tissue. Anisotropic conductivities in the normal myocardium were assigned values of 0.28 S/m in the longitudinal myofiber direction and 0.026 S/m in the transverse direction, which resulted in conduction velocity value of 45 cm/s in the longitudinal direction, matching swine conduction velocities reported by Vetter et al. (2005). Within the GZ, the transverse conductivity was decreased by 90% to represent connexin 43 remodeling, as has been done previously (Arevalo et al., 2013).

Membrane behavior in the normal tissue was represented by the Luo-Rudy dynamic model (LRd) (Luo and Rudy, 1994). The same membrane model was used, with modifications based on experimental data, to represent the electrophysiology of GZ cells, as we have previously described (Arevalo et al., 2013), namely: peak sodium current was decreased to 38% of the original value in the LRD model, peak L-type calcium current was decreased to 31% of the original value, peak potassium currents I_Kr_ and I_Ks_ were decreased to 30 and 20% of the original values, respectively. The modifications resulted in a decreased action potential upstroke velocity, decreased peak action potential magnitude, and increased action potential duration. Previous research by our team (Arevalo et al., 2013) has indicated that representation of the GZ as a homogeneously remodeled region provides an adequate approximation in the calculation of the morphology and location of the infarct-related reentrant circuits.

### Simulation protocol and data analysis

All simulations were performed using the software package CARP (CardioSolv, LLC) on a parallel computing platform (Vigmond et al., 2003; Plank et al., 2008). To examine the arrhythmogenic propensity of the post-MI ventricular models, programmed electrical stimulation (PES), similar to the protocol used in the pig experimental study, was simulated (Ashikaga et al., 2007). The models were paced from an epicardial location for 6 beats (S1) at a cycle length of 600 ms followed by a premature stimulus (S2) initially given at 90% of S1 cycle length. The timing between S1 and S2 was progressively shortened until VT was induced. If VT was not induced, a second premature stimulus (S3) was delivered after S2. If VT was not induced either, a third premature stimulus (S4) was delivered after S3. PES was performed at 10–12 epicardial sites to fully assess VT inducibility. Three seconds of VT was simulated.

Pseudo-ECGs were calculated in order to facilitate comparisons of global activation patterns during VT (Bishop and Plank, 2011). Two points separated by 20 cm were placed near the base of the heart with the line connecting them perpendicular to the base-apex axis. The difference in the extracellular potential was then calculated between the two points. To quantify difference between two ECG waveforms, the mean absolute deviation (MAD) metric was computed.

$$\text{MAD}=\frac{\sum_{i\;=\;1}^{n}\left|\left(X_{i}-\bar{X}\right)-\;(Y_{i}-\bar{Y})\right|}{\sum_{i\;=\;1}^{n}\left(\left|X_{i}-\bar{X}\right|+\left|Y_{i}-\bar{Y}\right|\right)}$$

Where *X* and *Y* are the pseudo-ECG waveforms of length n, and $\bar{X}$ and $\bar{Y}$ are the average values of the waveform. MAD varies between 0 and 100%, corresponding to identical and completely different waveforms, respectively.

**Figure 2:**
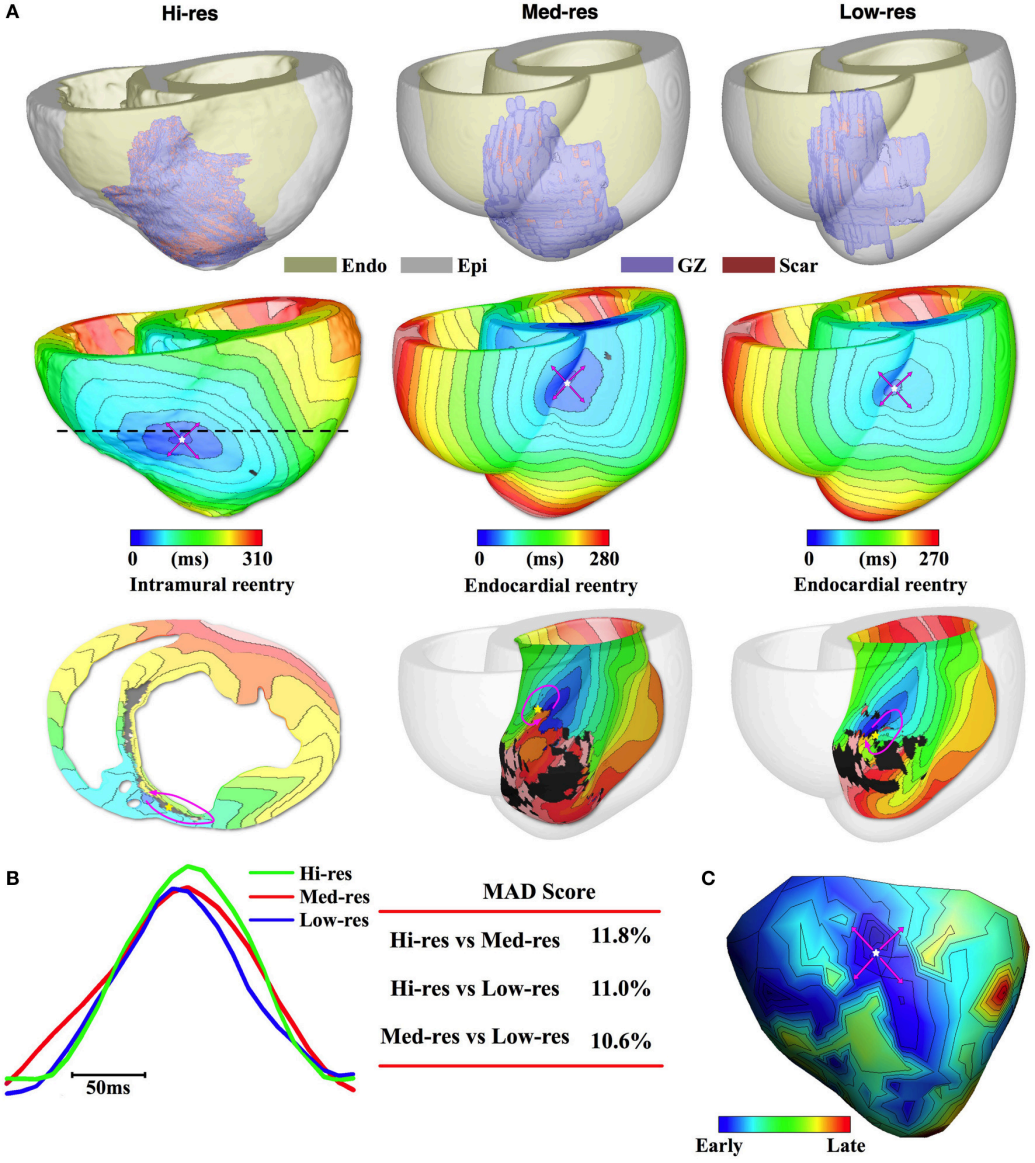
**Pig 1 simulation and experimental results. (A)** 1st row: Reconstructed models with the epicardium rendered semi-transparent. 2nd row: Activation maps of simulated VT all had breakthrough pattern on the epicardium. Pink arrows denote propagation direction. 3rd row: Intramural (Hi-res) or endocardial (Med-res and Low-res) view showing reentrant source. In the intramural view, the cross-section location is marked by dashed line in the 2nd row. **(B)** Pseudo-ECGs for one VT cycle length and the MAD score. **(C)** Experimentally recorded epicardial activation map.

**Figure 3:**
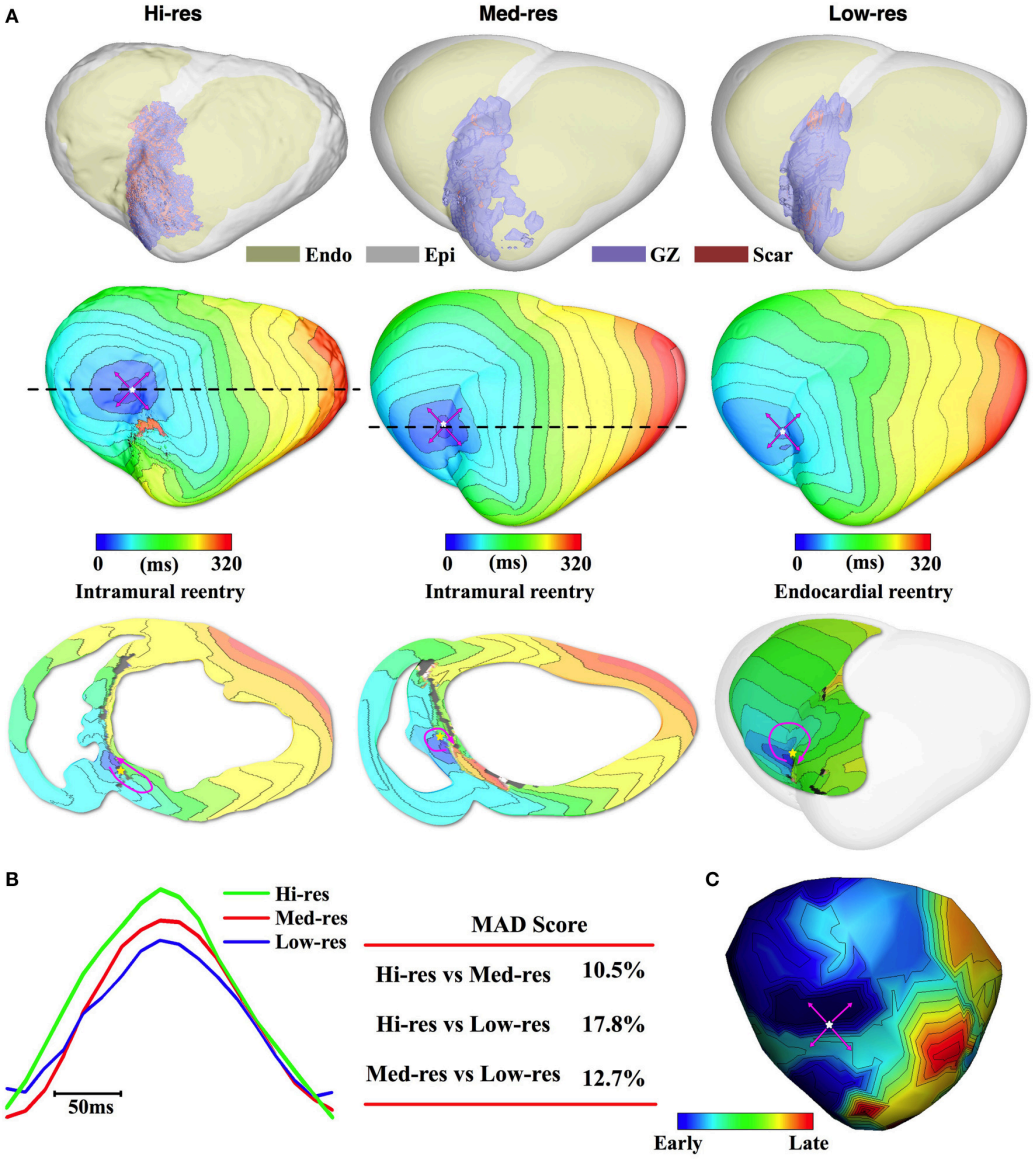
**Pig 2 simulation and experimental results. (A)** 1st row: Ventricular and infarct geometries with the epicardium rendered semi-transparent. 2nd row: Simulated VTs all had epicardial breakthrough pattern (Pink arrows: propagation direction). 3rd row: Intramural (Hi-res and Med-res) or endocardial (Low-res) view showing reentrant source. In the intramural view, the cross-section location is marked by dashed line in the 2nd row. **(B)** Pseudo-ECGs for one VT cycle length and the MAD score. **(C)** Experimentally recorded epicardial activation map had breakthrough pattern as well.

**Figure 4:**
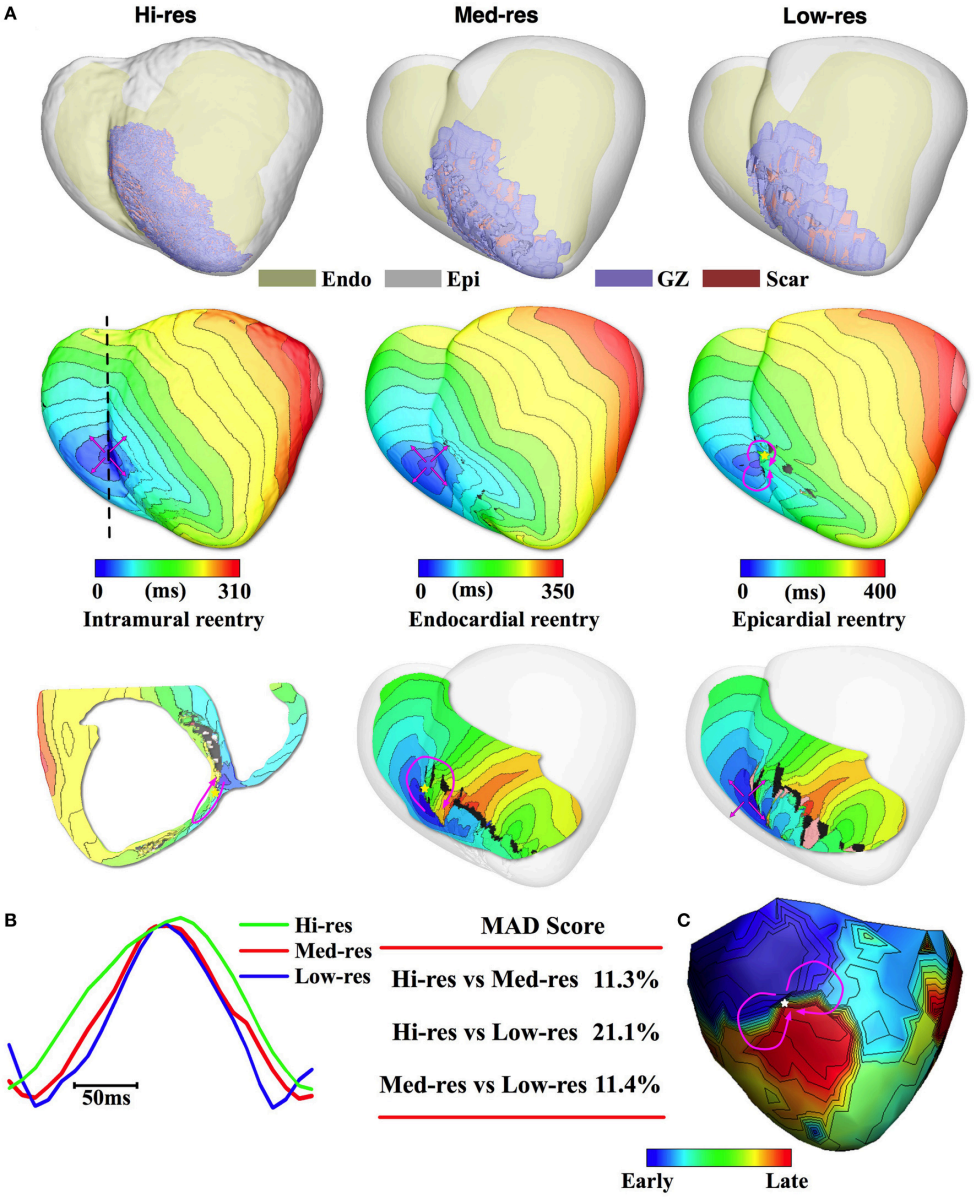
**Pig 3 simulation and experimental results. (A)** 1st row: Reconstructed models with the epicardium rendered semi-transparent. 2nd row: Activation maps of simulated VT had epicardial breakthrough pattern for the Hi-res and Med res models and epicardial figure-of-8 pattern for the Low-res model. Pink arrows denote propagation direction. 3rd row: Intramural (Hi-res) or endocardial (Med-res) view showing reentrant source. In the intramural view, the cross-section location is marked by dashed line in the 2nd row. For the Low-res model, endocardial view shows that epicardial reentry manifests as breakthrough on the endocardium. **(B)** Pseudo-ECGs for one VT cycle length and the MAD score. **(C)** Epicardial figure-of-8 activation pattern recorded during experimentally induced VT.

**Figure 5:**
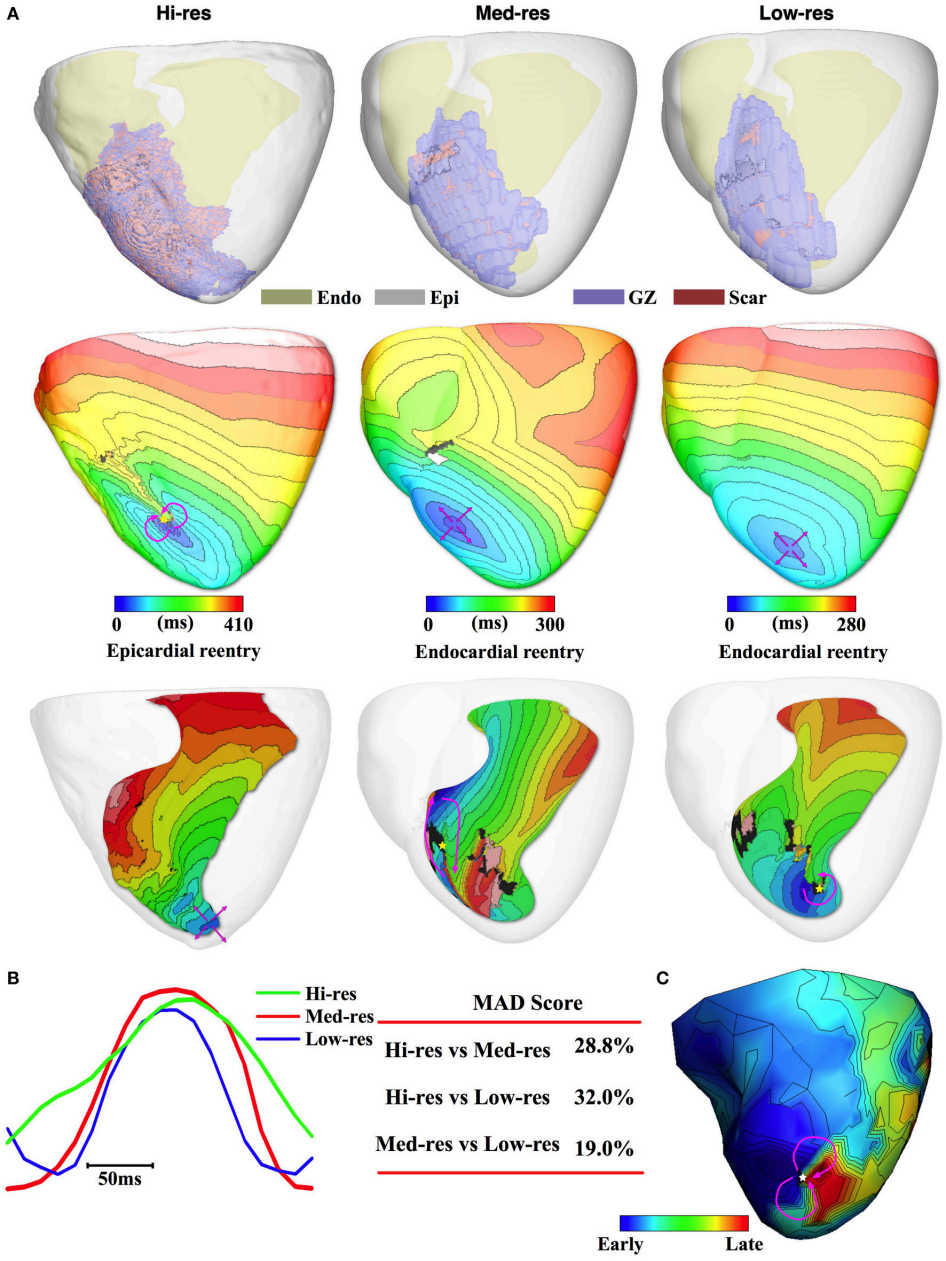
**Pig 4 simulation and experimental results. (A)** 1st row: Image-based models with different regions color coded and epicardium rendered semi-transparent. 2nd row: Simulated VT activation maps with epicardial figure-of-8 pattern for Hi-res and breakthrough for the downsampled models (pink arrows: propagation direction). 3rd row: Activation maps for LV endocardium showing how surface reentry manifests as breakthrough on endocardium (Hi-res model) and the reentrant source of epicardial breakthrough pattern (Med-res and Low-res models). **(B)** Pseudo-ECGs for one VT cycle length and the MAD score. **(C)** Activation map reconstructed from sock electrodes during an episode of induced VT had an epicardial figure-of-8 activation pattern.

Finally, the difference in location of the reentrant circuits induced in the different models was quantified. VT reentrant circuit location was estimated from the activation maps. In the case of reentrant circuits located on the epicardial or endocardial surface, the midpoint of the scar that anchors the reentry was identified (Figures 2–**5**, yellow star). If the epicardial VT pattern had a breakthrough morphology, intramural slices were used to locate the reentrant circuit and identify the scar that anchors the circuit.

## Results

### Accuracy of volume reconstructions

Table 1 summarizes the volumes of the normal myocardium, GZ, and scar in the reconstructed hearts. A paired Student's *t*-test revealed that there was no significance difference between total ventricular volumes between the Hi-res models (154.4 ± 7.5 cm^3^) and either of the Med-res or Low-res models (155.3 ± 9.4 and 157.9 ± 11.5 cm^3^, respectively). However, in all four hearts, the models reconstructed from the Med-res and Low-res images had significantly larger GZ and scar volumes compared to the Hi-res model (*p* < 0.05). Looking at the composition of each model, the Med-res and Low-res models had a significantly smaller percentage of normal myocardium (90.8 ± 0.8% and 92.0 ± 1.1%) compared to the Hi-res model (93.5 ± 1.5%). This demonstrated that the increase in GZ and scar volumes was due to expansion of the infarct onto normal tissue. This was to be expected since downsampling results in partial volume effect at the infarct periphery.

**Table 1:** **Normal, GZ, and scar volumes in the reconstructed heart models**.

		**Hi-res**		**Med-res**		**Low-res**	
		**Vol (cm^3^)**	**% Vol**	**Vol (cm^3^)**	**% Vol**	**Vol (cm^3^)**	**% Vol**
Pig1	Normal	139.7	94.0	150.5	91.0	155.4	91.7
	GZ	5.4	3.6	9.0	5.4	8.0	4.7
	Scar	3.5	2.4	5.8	3.6	6.0	3.5
Pig2	Normal	148.3	95.6	128.8	91.9	130.3	93.9
	GZ	4.3	2.8	8.1	5.8	7.7	3.3
	Scar	2.5	1.6	3.3	2.4	3.7	2.7
Pig3	Normal	153.9	92.5	139.5	89.9	148.1	91.4
	GZ	7.5	4.5	10.0	6.4	7.4	4.6
	Scar	4.9	2.9	5.7	3.7	6.5	4.0
Pig4	Normal	135.4	91.8	144.8	90.3	147.3	91.3
	GZ	8.2	5.6	9.9	6.2	9.6	5.9
	Scar	3.9	2.6	5.7	3.6	4.5	2.8
Mean ± SD	Normal	144.3 ± 7.2	93.5 ± 1.5	140.9 ± 8.0	90.8 ± 0.8	145.3 ± 9.2	92.0 ± 1.1
	GZ	6.4 ± 1.6	4.1 ± 1.0	9.3 ± 0.8	6.0 ± 0.4	7.4 ± 1.8	4.7 ± 0.9
	Scar	3.7 ± 0.9	2.4 ± 0.5	5.1 ± 1.1	3.3 ± 0.5	5.2 ± 1.1	3.3 ± 0.6
	Total	154.4 ± 7.5		155.3 ± 9.4		157.9 ± 11.5	

### Accuracy of VT simulations

For Pig 1, the ventricular geometry reconstructed from the different resolution images is presented in Figure 2A, 1st row. The overall structure of the anterior infarct in the Med-res and Low-res models matched well the Hi-res model. However, detailed structures in the infarct periphery, particularly near the apex, were lost as the image resolution decreased.

Despite the loss in infarct structure detail, the induced VT activation maps remained similar among the three models (Figure 2A, 2nd row). In the Hi-res model, PES resulted in the induction of a VT that manifested as breakthrough activity on the epicardium at the RV-septum junction of the anterior wall. The same epicardial breakthrough activity was induced in the Med-res and Low-res models. In the Hi-res model, a transmural slice through the epicardial breakthrough site (dashed line) revealed that the reentrant pathway driving VT was anchored to intramural scar located in the antero-septal region (Figure 2A, 3rd row). In the Med-res and Low-res models, the reentrant circuit was anchored to scar located on the LV-side of the septum endocardium. Despite the difference in the reentrant pathways, the distance between the scar that anchored the reentrant circuit in the Hi-res model and the scars that anchored the reentrant circuit in the Med-res and Low-res models was relatively close (13.6 and 9.4 mm, respectively, Table 2).

**Table 2:** **Distance between organizing centers of reentrant circuits**.

	**Hi-res to Med-res (mm)**	**Hi-res to Low-res (mm)**	**Med-res to Low-res (mm)**
Pig 1	13.6	9.4	10.3
Pig 2	14.5	18.3	4.5
Pig 3	6.2	7.9	2.4
Pig 4	13.0	9.4	17.1

The pseudo-ECGs, further demonstrated the accuracy of the downsampled models in predicting post-MI VT (Figure 2B). The representative traces during 1 VT cycle showed that the cycle length and overall morphology recorded from the three models were similar. This was evident in the low MAD score when the Hi-res trace was compared with the Med-res and Low-res traces (11.8% and 11.0%, respectively).

Finally, all three models accurately predicted the experimentally induced post-MI VT morphology. In the experiment, Pig 1 was inducible for VT with epicardial breakthrough (Figure 2C). The breakthrough was located on the anterior wall at the RV-septum border toward the base, similar to what was induced in all three models.

The simulation results for Pig 2 followed the same trend as the Pig 1 results. The 1st row of Figure 3A shows that the reconstructed models from the downsampled images accurately resolved the ventricular and infarct geometry. In all models, simulated PES resulted in induction of VT with breakthrough activity on the anterior epicardium (Figure 3A, 2nd row). In both the Hi-res and Med-res models, the underlying reentrant activity was an intramural circuit anchored to scar located in the septum (Figure 3A, 3rd row). The reentry-anchoring scar in the Med-res model was located 14.5 mm from the scar that anchored the reentry in the Hi-res model (Table 2). The close match in the reentrant patterns also resulted in a small MAD score (10.5%, Figure 3B). For the Low-res model, the breakthrough activity was driven by a reentrant circuit on the RV septum endocardial wall. The organizing center of this reentrant circuit was located 18.3 mm from the reentry-anchoring scar in the Hi-res model. The difference in activation pattern compared to the Hi-res model resulted in a slightly higher MAD score (17.8%) compared to the Med-res model. All three models successfully predicted the epicardial VT activation pattern and location of breakthrough site that was experimentally recorded in the same pig (Figure 3C).

Results for Pig 3 are presented in Figure 4; the ventricular and infarct geometry of the Hi-res model were again successfully resolved in the models reconstructed from the downsampled images (Figure 4A, 1st row). In the Hi-res model, PES resulted in the induction of breakthrough activity on the epicardium at the RV-septum junction (Figure 4A, 2nd row). The transmural view reveals that the underlying reentrant circuit was confined within the septum at the RV apex (Figure 4A, 2nd row). The epicardial activation map of the VT induced in the Med-res model, had a breakthrough pattern with the site of first epicardial activation identical to the one induced in the Hi-res model. The reentrant driver for this VT was located on the RV septum; whose organizing center was located 6.2 mm from the reentrant driver in the Hi-res model (Table 2). The similarity in the morphology and location of the reentrant circuit resulted in a low MAD score of 11.3% (Figure 4B). Meanwhile, in the Low-res model, PES resulted in the formation of a figure-of-eight reentrant circuit on the epicardium that manifested as breakthrough on the RV endocardium. While the VT morphology differed to what was observed in the Hi-res model, the Low-res model's reentrant circuit was still closely located to the reentrant circuit induced in the Hi-res model (7.9 mm). Interestingly, the simulated VT in the Pig 3 Hi-res model did not match the experimentally induced VT. In the experiments, Pig 3 was inducible for VT with a figure-of-8 epicardial reentry morphology (Figure 4C); the experimental pattern was matched best by the lLow-res model. Regardless, the location of the experimental reentrant circuit was close to the epicardial breakthrough and reentry site simulated in the Hi-res and Med-res models. Due to the difference in activation patterns, the Low-res model had a higher MAD score (21.1%) when compared to the Hi-res model.

Finally, the results for Pig 4 continue the trend of accurate geometric match between the three models observed in the other pigs (Figure 5A, 1st row). In this pig, PES in the Hi-res model resulted in the induction of a figure-of-eight reentrant pattern on the epicardium located on the anterior RV-septal junction toward the apex (Figure 5A, 2nd row) and manifests as breakthrough activity on the LV endocardial surface (Figure 5A, 3rd row). On the other hand, the Med-res and Low-res models resulted in the induction of breakthrough activity on the epicardium with the epicardial breakthrough located near the figure-of-8 reentry site induced in the Hi-res model. In both Med-res and Low-res models, the epicardial breakthrough was driven by rotors located on the LV endocardium. The Med-res and Low-res rotors were located closely to the High-res rotor (13.0 and 9.4 mm, respectively). Due to difference in the VT morphologies between the Hi-res model and the Med-res and Low-res models, the MAD score for Pig 4 was higher than the ones calculated for the other pigs (28.8 and 32.0%, respectively, Figure 5A, 3rd row).

The experimental recording revealed that Pig 4 was inducible for figure-of-8 reentry located at the anterior apex (Figure 5C). The simulation results of the Hi-res model successfully matched the experimentally induced VT pattern and location. Meanwhile the Med-res and Low-res models were unsuccessful in accurately predicting the experimental VT morphology, but the two models were still able to accurately identify the location of the arrhythmogenic substrate in the heart.

## Discussion

In this work, we successfully created image-based models of post-MI ventricles reconstructed from both high-resolution *ex-vivo* MRI and as well as from clinical MRI scans. Simulations with these image-based models were used to predict the post-MI arrhythmogenic reentrant circuits following PES. While there have been previous attempts in simulating post-MI electrophysiological activity (Vigmond et al., 2009; Pop et al., 2011; Ng et al., 2012), this is the first study to use experimentally obtained VT activation maps to validate model predictions as well as demonstrate that the locations of the *in-silico* predicted VT circuits are fairly well preserved in models reconstructed from low resolution MRI. These results pave the way for the translation of *in-silico* modeling of post-MI arrhythmogenesis into clinical utility.

Previous experimental and clinical studies have identified the heterogeneous structure of infarcted tissue as the arrhythmogenic substrate in post-MI hearts. Specifically, studies have shown that the presence of partially viable tissue interdigitated with electrically inert scar promote conduction slowing and provide pathways that could harbor reentrant circuits (Stein et al., 2008; Suk et al., 2008). The experiments that produced the retrospective pig data used in the current study were the first to combine high resolution *ex-vivo* CE-MRI with multi-electrode sock recording in order to determine the relationship between VT circuits and specific infarct structure (Ashikaga et al., 2007). The results demonstrated that islands of viable myocardium within the scar correlated with epicardial breakthrough sites in the case of centrifugal VT pattern and with the isthmus in the case of epicardial reentry VT pattern. While this study hinted at the utility of MRI-based scar analysis to predict the location of the arrhythmogenic substrate, the image resolution for *ex-vivo* preparations is currently not achievable in the clinical setting.

We demonstrate in this study that the complexity of infarct structure can be reasonably reconstructed from low resolution images. In all four pigs, the models reconstructed from both the Med-res and Low-res images had infarct geometries similar to that obtained from the Hi-res *ex-vivo* images. In the infarct periphery, downsampling of the images led to partial volume effect leading slight overestimation of the infarct volume as compared to the Hi-res images. There was also difficulty in resolving infarct structure close to the apex. This was due to uneven diffusion of gadolinium toward the apex resulting in decreased maximum image intensity close to the apex (Schelbert et al., 2010). Despite this loss in infarct structural detail, the overall infarct architecture was preserved in the downsampled images. Additionally, the percent composition of scar and GZ in the infarct was comparable between the Hi-res images and the downsampled images.

Simulations with the reconstructed hearts demonstrated the accuracy of the models in predicting the location of infarct-related reentrant circuits. In the Hi-res models, three of the four hearts successfully predicted the epicardial morphology of the experimentally obtained VT in the *ex-vivo* hearts. In all four hearts, the simulations successfully predicted the location of the reentrant circuit or the epicardial breakthrough site. In the three hearts with epicardial breakthrough morphology, the simulations revealed that the underlying reentrant circuit was located intramurally or on the endocardium. This was consistent with the Ashikaga et al's findings that swine hearts were more likely to have endocardial than epicardial reentrant circuits (Ashikaga et al., 2007).

Using the simulation predictions from the Hi-res models as ground truth, the predicted VT location remained fairly accurate for the models reconstructed from the downsampled images. The VT activation maps in the Med-res and Low-res models matched the morphology of the epicardial activation recorded in 75 and 50%, respectively, of the Hi-res pig hearts. In order to quantitatively measure the difference of electrical conduction between different images resolution, we use MAD score of the pseudo-ECGs (Vadakkumpadan et al., 2012). The higher overall accuracy of the Med-res models compared to the Low-res models was reflected in the lower mean MAD scores (15.6 ± 7.6 vs. 20.5 ± 7.6%). In any case, the relatively low MAD scores for both Med-res and Low-res models demonstrate the accuracy of the downsampled models in simulating overall VT activation patterns.

The downsampled models had a higher success in predicting the location of the reentrant circuits. The predicted reentrant circuits in the Med-res and Low-res models were closely located from the circuits induced in the Hi-res models (11.8 ± 3.3 vs. 11.3 ± 4.1 mm). Since current radiofrequency ablation lesions can have up to 14 mm width (Petersen et al., 1999), the predicted circuits in the downsampled models were within one ablation lesion away from the “true” VT reentrant circuit location predicted in the Hi-res models. Since clinical ablation usually involve the delivery of multiple lesions in one area, using models reconstructed from clinical images to guide VT ablation appears feasible.

The results of this study could have implications for the potential use of patient-specific simulations of infarct-related VT in predicting the optimal sites of VT ablation. The relatively high level of preservation of the locations of the organizing centers of reentry or sites of breakthrough upon downsampling of the images, as found here, indicates that it might be feasible to use patient-specific heart models reconstructed from clinical MR scans to determine the VT morphology in the given patient and thus to provide guidance as to what ablation lesions would best terminate VT. Future studies are needed to address this subject of major clinical significance.

## Limitations

A limitation of the current study is the small sample size of only four pig hearts. The original experimental study included more pigs but a few had chaotic epicardial VT patterns, indicative of complex intramural reentrant activity, which was difficult to match *in silico*. Several of the pigs underwent endocardial basket electrode recording which was difficult to register with the MRI scan. Purkinje fibers were also not included in the models which might have affected the propagation patterns during VT, especially at sites distal from the reentry origin. Finally, some of the *ex-vivo* images had signal noise due to gadolinium leak. In these cases, the images had to be manually cleaned.

## Funding

NIH Pioneer Award (DP1-HL123271) to NT.

### Conflict of interest statement

Dr. Natalia Trayanova serves on the scientific advisory board of CardioSolv, LLC. The other authors declare that the research was conducted in the absence of any commercial or financial relationships that could be construed as a potential conflict of interest.
